# Protein trafficking, ergosterol biosynthesis and membrane physics impact recombinant protein secretion in *Pichia pastoris*

**DOI:** 10.1186/1475-2859-10-93

**Published:** 2011-11-03

**Authors:** Kristin Baumann, Núria Adelantado, Christine Lang, Diethard Mattanovich, Pau Ferrer

**Affiliations:** 1 Department of Chemical Engineering, Universitat Autònoma de Barcelona, Bellaterra (Cerdanyola del Vallès), Spain; 2 Department of Biotechnology, Technical University of Berlin, Berlin, Germany; 3 Department of Biotechnology, University of Natural Resources and Life Sciences, Vienna, Austria; 4 Austrian Centre of Industrial Biotechnology (ACIB GmbH), Vienna, Austria

## Abstract

**Background:**

The increasing availability of 'omics' databases provide important platforms for yeast engineering strategies since they offer a lot of information on the physiology of the cells under diverse growth conditions, including environmental stresses. Notably, only a few of these approaches have considered a performance under recombinant protein production conditions. Recently, we have identified a beneficial effect of low oxygen availability on the expression of a human Fab fragment in *Pichia pastoris*. Transcriptional analysis and data mining allowed for the selection of potential targets for strain improvement. A first selection of these candidates has been evaluated as recombinant protein secretion enhancers.

**Results:**

Based on previous transcriptomics analyses, we selected 8 genes for co-expression in the *P. pastoris *strain already secreting a recombinant Fab fragment. Notably, *WSC4 *(which is involved in trafficking through the ER) has been identified as a novel potential target gene for strain improvement, with up to a 1.2-fold increase of product yield in shake flask cultures. A further transcriptomics-based strategy to modify the yeast secretion system was focused on the ergosterol pathway, an aerobic process strongly affected by oxygen depletion. By specifically partially inhibiting ergosterol synthesis with the antifungal agent fluconazole (inhibiting Erg11p), we tried to mimic the hypoxic conditions, in which the cellular ergosterol content was significantly decreased. This strategy led to an improved Fab yield (2-fold) without impairing cellular growth. Since ergosterol shortage provokes alterations in the plasma membrane composition, an important role of this cellular structure in protein secretion is suggested. This hypothesis was additionally supported by the fact that the addition of non-ionic surfactants also enhanced Fab secretion.

**Conclusions:**

The current study presents a systems biotechnology-based strategy for the engineering of the industrially important yeast *P. pastoris *combining the use of host specific DNA microarray technologies and physiological studies under well defined environmental conditions. Such studies allowed for the identification of novel targets related with protein trafficking and ergosterol biosynthesis for improved recombinant protein production. Nevertheless, further studies will be required to elucidate the precise mechanisms whereby membrane biogenesis and composition impact on protein secretion in *P. pastoris*.

## Background

The combination of the unexpectedly fast progress in genome sequencing over the last decade and 'omics' analytical platforms have provided an invaluable source of information on the physiology of yeasts, including a comprehensive overview on different cellular processes. In several genome scale studies, *Saccharomyces cerevisiae *has served as a useful model system to explain the complexity of stress responses at the transcriptome level, comprising important factors like temperature [1-3], nitrogen starvation [4-6], osmolarity [7] and oxygen availability [8-11]. Nevertheless, only a small number of such studies have investigated the impact of environmental perturbations on already engineered yeast strains (reviewed in [12]) - a scenario which is very likely to resemble industrial processes. Considering the relevance of yeast cell factories for commercial purposes and the tight interrelation between environmental stresses and protein folding and secretion, such comprehensive studies are currently emerging as promising platforms for systematic yeast strain engineering. For instance, transcriptomic studies of recombinant *S. cerevisiae *expressing a membrane protein [13] have lead to the construction of improved production strains based on the over expression of *BMS1*, involved in ribosome biogenesis, or deletion of several genes involved in transcriptional regulation [14]. Sauer and co-workers [15] reported the first series of genome scale cell physiology studies of recombinant *P. pastoris *under stress conditions. They compared the transcriptional profile of a recombinant *P. pastoris *strain expressing human trypsinogen to that of a non-expressing strain. Based on the outcome of that work, Gasser et al. [16] selected a range of significantly regulated genes and tested their *S. cerevisiae *homologues for co-expression in a recombinant *P. pastoris *strain. Back then, the identification of six novel (*BMH2*, *BFR2*, *SSA4*, *SSE1*, *CUP5 *and *KIN2*) and five previously described secretion helpers (*PDI1*, *ERO1*, *HAC1*, *KAR2 *and *SSO2*) already pointed to the success of such a strain engineering strategy. The recent publication of the *P. pastoris *genome sequence [17,18] permitted the development of host-specific microarrays [19], and with that also the independency of data interpretation on similarities in *S. cerevisiae*. Dragosits and co-workers recently reported the first *P. pastoris *specific 'omics' studies by investigating the effect of temperature [20] and osmolarity [21] on the transcriptome, proteome and metabolic fluxes in a recombinant strain secreting an antibody Fab fragment. Their work uncovered a decrease of the protein folding stress at lower temperatures and, therefore, a possible correlation with the beneficial effect on protein secretion. High osmolarity, on the other hand, did not affect product yield, but trigged a physiological response different from that described for *S. cerevisiae*. Data generated from these studies certainly form a useful knowledge base for future systems metabolic engineering studies, providing not only information on environmental stress regulations under protein production conditions, but also new insights into both host-specific and common limitations in the secretion system when compared with other expression platforms.

In an analogous study, we have recently reported the transcriptome, proteome and fluoxome of a recombinant *P. pastoris *strain expressing a Fab antibody fragment under three different conditions of oxygen availability [22]. Paradoxically, although the need of sufficient oxygen for oxidative protein folding and other processes would be expected, we observed a beneficial effect of hypoxic conditions on the specific productivity in chemostat cultivations as well as in fed batch fermentations [23]. This striking observation encouraged a more detailed analysis of 'omics' data aiming at the identification of potential new targets for strain improvement. The outcome of this data mining study and direct application of potential candidates in screening experiments are presented in this work.

## Results

### Identification and cloning of potential target genes for strain improvement

In order to select potential target genes for strain improvement of the yeast *P. pastoris*, we compared the mRNA profile of a recombinant strain to that of a non-expressing control strain grown under different oxygenation conditions [22]. This recombinant 'reference strain' secreted the 3H6 antibody Fab fragment as model recombinant protein complex [24] and showed an increased secretion capacity under hypoxic growth conditions [23]. Target genes for improved Fab expression were mainly selected on the basis of the magnitude of their regulation (relative ratios, exceeding the fold change threshold of ± 1.5) in the recombinant strain between hypoxic and normoxic conditions, as well as on their potential relevance for recombinant protein production. In addition, we included two genes that were significantly up regulated in the recombinant strain as compared to the non-producing control strain under hypoxic conditions.

Overall, eight potentially interesting genes were considered for further analysis of the impact of their overexpression on recombinant protein secretion (see Table 1 for details).

**Table 1:** List of target genes for co-expression in *P.pastoris *Fab 2F5

Target gene	ORF	*Description*	fold change	adj p-value
**ERO1**	PIPA00063	*Thiol oxidase required for oxidative protein folding in the endoplasmic reticulum*	6.49	9.15E-07

NCE103 *	PIPA03864	*Carbonic anhydrase, involved in non-classical protein export pathway*	3.36	4.08E-08

AQR1 *	PIPA04502	*Plasma membrane multidrug transporter of the major facilitator superfamily*	17.03	2.25E-09

**SLY41**	PIPA02527	*Protein involved in ER-to-Golgi transport*	1.85	3.22E-04

**TDH3**	PIPA02510	*Glyceraldehyde-3-phosphate dehydrogenase, involved in glycolysis and gluconeogenesis*	4.38	5.44E-06

**TEF4**	PIPA00834, PIPA10574	*Gamma subunit of translational elongation factor eEF1B, stimulates the binding of aminoacyl-tRNA to ribosomes*	36.25	6.15E-10

**TSA1**	PIPA04168	*Thioredoxin peroxidase, self-associates to form a high-molecular weight chaperone complex under oxidative stress*	5.78	2.83E-06

**WSC4**	PIPA00592	*ER membrane protein involved in translocation of soluble secretory proteins and insertion of membrane proteins into the ER membrane*	2.57	8.82E-05

Target genes (systematic names and *P. pastoris *specific PIPA codes) for co-expression are listed together with their biological function. The fold change ratios and adjusted *p*-values derive from the pairwise comparison of hypoxic and normoxic conditions. Target genes highlighted with (*) are also significantly (*p*-value < 0.05) upregulated in the Fab expressing strain compared to the control strain in hypoxic conditions.

Genes for co-overexpression were amplified from *P. pastoris *genomic DNA and transformed into a *P. pastoris *X-33 strain expressing a 2F5 Fab antibody fragment under control of the glycolytic GAP promoter. In this study, the Fab 2F5 was used as a model protein for screening because the ELISA assay used for quantification of the Fab titer was originally optimized for this antibody and showed a higher reproducibility with Fab 2F5 than with its anti-idiotypic Fab 3H6 which was used in the genome-scale study. Furthermore, such strategy emerging from more than one model protein may also indicate a broader applicability of the results.

Twelve individual clones of each co-overexpressing strain were verified for integration of the gene of interest by PCR. Some of the clones were lacking the insert, thereby reducing the number of transformants for further screening studies in 24-well culture plates to eight clones per target gene construct.

### The effect of co-expressed target genes on recombinant Fab secretion in *Pichia pastoris *24-well and shake flask cultures

**Figure 1:**
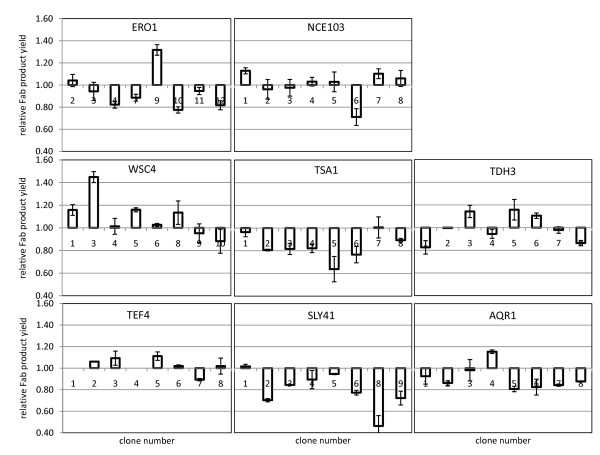
**24-well plate screening of co-expressing strains**. Eight individual clones of each target gene and of a control strain containing the empty vector were used for a small scale screening in 24-well plates. All cultures started with the same initial OD_600 _of 0.1 in BMD medium containing 2% (w/v) glucose. After 24 hours, Fab titers and wet cell weight were calculated to determine the product yield (mg_Fab _g_WCW_^-1^) at a given cultivation point (24 h). The yield ratios relative to the control strain (value = 1) are illustrated. Error bars indicate the standard error of the means.

Series of eight verified clones overexpressing one of the target genes of interest were used in a first small scale screening in 24-well culture plates in duplicate experiments and with random positions of the clones on the 24-well plate. Product titers and biomass were measured by ELISA and wet cell weight quantification, respectively. The average Fab yields were normalized to those obtained from the empty vector control strain, which showed very uniform expression levels also between clones (data not shown). The results of these preliminary screening experiments are shown in Figure 1. The overall picture of co-expression of the selected target genes demonstrated a largely unchanged protein secretion capacity, even indicating disadvantageous rather than beneficial effects for several target genes. Mutants with an essentially uniform negative impact on Fab yield were those co-expressing *TSA1*, *SLY41*, *AQR1 *and, intriguingly, *ERO1*, which has been previously reported to enhance protein secretion in *S. cerevisiae *[25] as well as in *P. pastoris *[16]. Only one *ERO1 *mutant (*ERO1 *clone #9) seemed to favour Fab secretion significantly in this small-scale format. No such well-defined outcome was observed with the mutants of *NCE103*, *TEF4 *and *TDH3*, since most of them did not show any clear effect on Fab yield (neither positive nor negative). Only clones co-expressing *WSC4 *demonstrated more promising results, with approximately 50% having a beneficial effect on Fab yield. Among these tested transformants there was one *WSC4 *mutant (*WSC4 *clone #3) with a prominent increase in specific Fab expression (1.45 fold) relative to the control strain.

**Figure 2:**
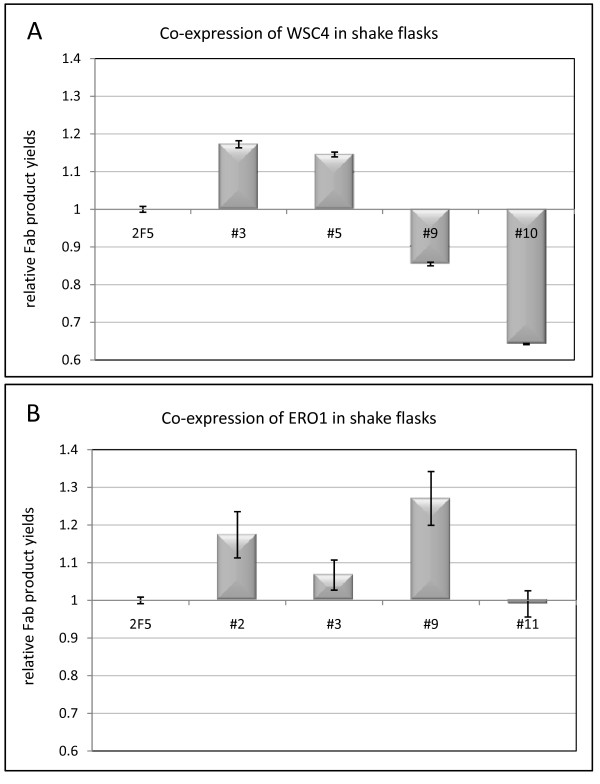
**Shake flask screening of clones co-expressing *ERO1 *and *WSC4***. Relative product yields of *ERO1 *(**A**) and *WSC4 *(**B**) co-expressing *P. pastoris *clones (4 individual clones from each target gene) secreting the recombinant Fab antibody. The data were normalized to the control culture values, and error bars indicate the standard error of the means.

The Fab secretion capacity of a reduced set of ERO1 and WSC4 clones (# 2, 3, 9, 11 and # 3, 5, 9, 10, respectively), was further verified by extending the number of biological replica to three and performing cultivations on a larger scale, that is, using baffled shake flasks. The outcome of these experiments is shown in Figure 2. The overall expression profile obtained and, in particular, the recombinant Fab secretion increase of about 1.2-fold for both ERO1 clone #9 and WSC4 clone #3, confirmed the results obtained in 24-well plates cultivations.

A plausible explanation for the observed clonal variation would be that isolated transformants differed in the dosage of the co-expressed *ERO1 *and *WSC4 *genes. In order to test this hypothesis, the relative dosage and transcriptional levels of *ERO1 *and *WSC4 *were determined by qPCR for two independent clones of each construct (ERO1 clones #9 and 11, and WSC4 clones #3 and 10), one giving a clear increased Fab yield and the other no significant (or negative) effect on product yield, compared with the reference strain. Results revealed that there was no difference in the co-expressed gene dosage. Moreover, differences in terms of mRNA levels for *ERO1 *and *WSC4 *genes amongst the corresponding selected clones were not statistically significant. Also, Fab LC and HC mRNA levels amongst these clones, as well as with respect to the reference strain, were similar (data not shown). This suggested that other parameters may be involved in the clonal variation observed in terms of Fab secretion yield.

### Identification and manipulation of potential target metabolic pathways for strain engineering

**Figure 3:**
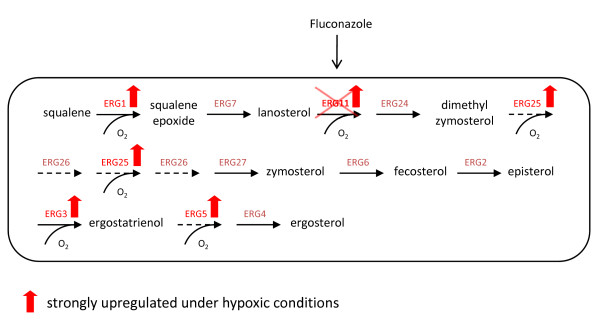
**Schematic view of the ergosterol pathway and fluconazole inhibition**. Outline of the post-squalene ergosterol biosynthetic pathway illustrating the step inhibited by fluconazole. Dashed arrows indicate no specification of intermediates, and red arrows highlight the genes that are upregulated in the Fab strain in hypoxic conditions.

In addition to the identification of novel target genes involved in cellular processes directly connected to protein secretion, transcriptomic analyses allowed the identification of other metabolic pathways with notable alterations in hypoxic conditions and, therefore, potentially interfering or related to protein secretion. In particular, we focused on the ergosterol and sphingolipid synthesis pathways, which are aerobic processes requiring molecular oxygen and, therefore, were particularly affected under hypoxic conditions. For both, Fab-producing and empty vector control strains, the regulation pattern was very uniform, with a considerable induction of a number of genes catalyzing oxygen dependent reactions. A specific observation that prompted us to investigate in the first place one these pathways in more detail was the significantly stronger induction of the ergosterol biosynthesis gene *ERG25 *in the recombinant strain when compared to the non-expressing strain. A scheme of the ergosterol synthesis pathway is illustrated in Figure 3. It is well known that manipulating the ergosterol pathway can be delicate, especially in terms of deletion mutants, since many of the ERG genes are essential. The first strategy to circumvent this problem was the treatment with the antifungal agent fluconazole, a specific inhibitor of lanosterol C-14alpha demethylase encoded by *ERG11*, as indicated in Figure 3.

A second approach was the addition of detergents to the culture medium. These compounds, in particular Tween 80, are typically supplied to anaerobic *S. cerevisiae *cultivation media in order to sustain growth. Furthermore, such non-ionic surfactants are also known to alter the membrane fluidity of the cells [26,27]. Since ergosterol is an important component of the yeast membrane and alterations in sterol distribution (i.e. by inhibition of ergosterol synthesis) are known to impact membrane permeability [28], application of detergents like Tween or Triton might exert a similar effect.

### Effect of partial inhibition of the ergosterol pathway on heterologous protein secretion - treatment with Fluconazole

**Figure 4:**
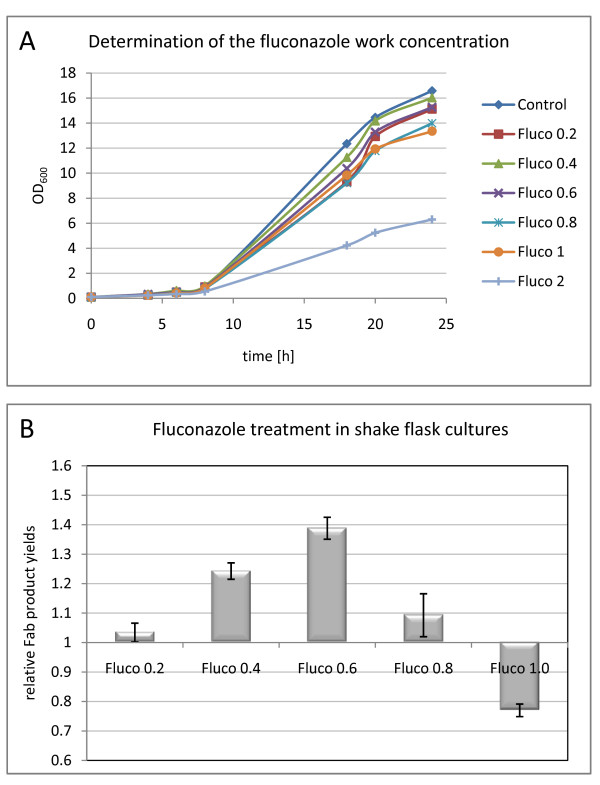
**Fluconazole screening**. A: Determination of the fluconazole working concentrations. Growth curves of the recombinant *P. pastoris *strain X-33 pGAPZαA Fab2F5 grown in BMD medium and in the presence of 0 - 2 μg ml^-1 ^fluconazole. B: Fluconazole screening in shake flask cultures. Fab productivities in different concentrations of fluconazole (0.2 - 1.0 μg ml^-1^) were normalized to the values obtained from non-treated cells. The relative Fab productivities for each concentration are demonstrated, error bars indicate the standard error of the means.

The *P. pastoris *2F5 Fab expressing strain was cultivated in 24-well plates in the presence of 0~100 μg ml^-1 ^fluconazole. After plotting the growth curves (Figure 4A), we determined a range between 0.2 and 1 μg ml^-1 ^to be optimal for the fluconazole screening experiments. 1 μg ml^-1 ^turned out to be the maximum concentration without showing any interference with cellular growth. The screening in baffled shake flasks included biological triplicates for concentrations of 0.2, 0.4, 0.8 and 1.0 μg ml^-1^, as well as the negative control without fluconazole treatment. As shown in Figure 4B, fluconazole in concentrations up to 0.6 μg ml^-1 ^had a beneficial effect on Fab secretion (about 1.4-fold increase), whereas higher concentrations lead to a negative effect.

### Sterol analyses after Fluconazole treatment

Since fluconazole inhibits the lanosterol C-14α demethylase step in the ergosterol biosynthesis pathway, the treatment with this antifungal drug is expected to decrease the ergosterol content. In order to prove this hypothesis, sterol composition from cells grown in the presence of 0.6 μg ml^-1 ^fluconazole, that is, the concentration in which a higher Fab secretion was achieved, was analyzed and compared to the sterol content of non-treated cells (Table 2). Indeed, results summarized in Table 2 show a different sterol composition when comparing fluconazole-treated vs. untreated cells, including a clear decrease in the ergosterol content. Notably, the analyses also revealed the appearance of two novel sterol compounds in the fluconazole-treated cells, which could not be identified by GC-MS (see Supplementary file 1). These compounds, which showed a higher retention time than ergosterol in GC analyses, could be intermediates of the ergosterol biosynthesis pathway, e.g. lanosterol. In fact, previous studies [29] have shown that inhibition by of sterol-14α-demethylase by fluconazole (and other azole drugs) results in depletion of ergosterol and accumulation of the substrate, lanosterol, as well as 14α-methylated sterols.

**Table 2:** Composition of major sterols in both untreated and fluconazole-treated *P. pastori s *cells

Sterol	μg Sterol/mg DCW	
	
	Control culture	Fluconazole-treated culture
Unknown 1	0	0.5
Unknown 2	0	0.47
Squalen	0	0
Zymosterol	0.17	0
**Ergosterol**	**9.81**	**6.77**
Fecosterol	0.21	0
Episterol	0.23	0

**Total sterols**	**10.42**	**7.74**

Amounts of sterol (μg_sterol _mg_DWC_^-1^) were determined from *P. pastoris *X-33 pGAPZαA Fab2F5 cells treated with 0.6 μg ml^-1 ^fluconazole and compared with untreatred cells. Unknown 1 and 2 refer to non-identified compounds detected in GC analyses of the sterol fraction of fluconazole-treated *P. pastoris *cells (see Additional file 1).

### Effect of potential alterations in membrane fluidity by treatment with non-ionic surfactants

**Figure 5:**
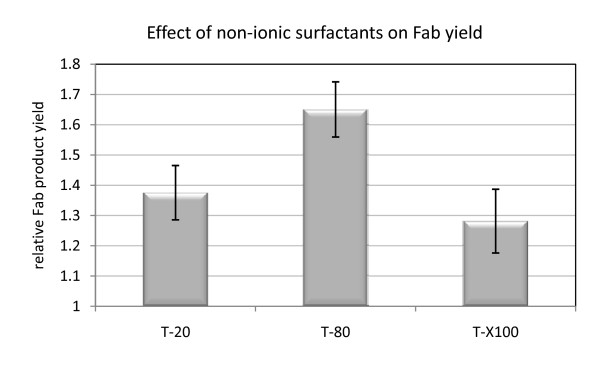
**Effect of Tweens and Triton X-100 on Fab yield in shake flasks**. The effect of the non-ionic surfactants Tween 20 (T-20), Tween 80 (T-80) and Triton X-100 (TX-100) on the Fab productivity in *P. pastoris *X-33 pGAPZαA Fab2F5 is demonstrated. The mean ratios of treated samples normalized to the untreated control samples are illustrated, and error bars indicate standard errors of the means.

In preliminary experiments, we identified Tween 80 as enhancer for protein secretion, increasing the product yield 3-fold in small scale 24-well plate cultivations (data not shown). As a consequence, a more detailed analysis of the effect of Tween 80 and two closely related non-ionic surfactants - Tween 20 and Triton X-100 - on recombinant protein secretion was performed in shake flasks cultures of the *P. pastoris *X-33 2F5 Fab expressing strain. All three detergents were added at a concentration of 42 mg l^-1^, which is typically applied to anaerobic *S. cerevisiae *cultivations (see for example [30]). Tween 80 addition to shake flask cultures increased Fab secretion 1.65 fold. A stimulating effect could also be seen with Tween 20 (1.3 fold) and Triton X-100 (1.4 fold) (Figure 5).

## Discussion

Systems biotechnology offers new strategies for yeast strain engineering [31]. In this study, a DNA microarray based data mining from experiments that showed an increased productivity of a complex foreign protein in *P. pastoris *has led to the identification of novel target genes or pathways for the improvement of heterologous protein production.

Engineering of the protein folding and quality control machinery is a very common and successful strategy to increase the secretory capacity of yeasts. For instance, overexpression of the unfolded protein response (UPR) proteins Pdi1p and Hac1p, the ubiquitin Ubi4p, and the chaperones BiP/Kar2p, Jem1p and Cne1p have been extensively shown to improve protein secretion (all reviewed in [12]).

The protein disulfide isomerase (Pdi) recycling assistant Ero1p, an ER membrane-resident protein and key component of the oxidative folding machinery, has also been reported as helper factor to increase the yields of human serum albumin in *K. lactis *[32] and of the human Fab 2F5 antibody fragment in *P. pastoris *[16]. It is particularly for this reason that we expected to observe a similar effect in this study. At this point, however, it has to be stated that although the model protein in this study was identical to that used by Gasser and co-workers [16], the experimental design differed in three pivotal points: firstly, the strain was a protease deficient SMD1168 (*pep4 *mutant); secondly, the expression vector carrying the gene *ERO1 *was based on histidine selection, 3 times bigger in size and integrated in a different locus (*HIS4*); and thirdly, the *ERO1 *gene was derived from *S. cerevisiae *genomic DNA, while in this study the host specific *P. pastoris ERO1 *gene was amplified and over expressed. Despite these dissimilarities, the magnitude of the beneficial effect shown by *ERO1 *clone#9 was overlapping with that reported in Gasser et al. [16]. In relation to the clonal variation observed amongst ERO1 clones (as well as for the rest of co-expressed genes), it has recently been observed that the gene dosage of *PDI *determines whether its co-expression has a beneficial or detrimental impact on foreign protein secretion [33]. Besides, clonal variation could be also partially due to genetic differences (mutations) generated during the transformation event, as previously suggested [34].

Unlike *ERO1*, *WSC4 *has not been described as a helper factor for increased protein secretion so far. Wsc4p is a component of the ER and plays a role in the translocation of soluble proteins as well as the insertion of proteins targeted to the ER membrane. A contribution of the WSC family to enhanced environmental stress resistance has also been suggested [35,36]. Interestingly, *WSC4 *is the closest of the four *S. cerevisiae *homologues to the *TSR1 *gene of *Yarrowia lipolytica*, whose gene product assists in the signal recognition particle (SRP) - dependent translocation of proteins through the ER and also interacts with the UPR-regulator BiP [37]. The potential link to the UPR and its role in protein sorting through the ER membrane may explain the beneficial effect of *WSC4 *overexpression on Fab secretion. Consistent with these results is the finding that a *S. cerevisiae *null mutant of *WSC4 *(the gene is termed *YCH8*) accumulated soluble protein precursors, indicating defects in protein trafficking [38]. A further link between the WSC family and the secretory pathway includes the activity of Wsc1p in secretory defective cells, where it is required for the repression of genes that make up the protein-synthetic machinery [39]. Although this observation would contradict the herein proposed role of *WSC4 *on protein secretion (inferred from sequence similarities), it has to be stated that that the functions of the Wsc proteins overlap only partially. In fact, an implication in the signalling pathway that mediates the response to an interruption of the secretory pathway has been reported also for Wsc2p and Wsc3p, but not for Wsc4p [40]. We therefore believe that *WSC4 *deserves closer attention as a potential target of the secretory system for engineering host cells.

During the identification of potential target genes significant changes were observed in the oxygen-dependent ergosterol and sphingolipid biosynthesis pathways. In addition to the strong transcriptional induction of these pathways in both strains under hypoxic conditions and, in particular, of oxygen consuming reactions therein, *ERG25 *was also significantly stronger expressed in the Fab producing strain. Considering that this stronger induction resulted not only from oxygen depletion but also from the additional charge of recombinant protein expression, we suggested a potential role of ergosterol or an intermediate during the protein secretion process. In order to investigate the capacity of this important component of the yeast membrane to influence protein secretion, its biosynthesis was perturbed by applying the antifungal agent fluconazole, which specifically inhibits the activity of Erg11p in the late steps of the ergosterol pathway (see Figure 3). Interestingly, the cumulative silencing of its synthesis by applying increasing amounts of fluconazole favoured protein secretion to its maximum at a concentration of 0.6 μg ml^-1^, while higher concentrations inhibited growth and therefore suggest a complete breakdown of ergosterol synthesis with fatal consequences for the cell. This partial silencing of the ergosterol synthesis by inhibiting Erg11p may resemble the ergosterol deprivation in hypoxic conditions. In fact, the ergosterol content was significantly decreased in fluconazole-treated cells, as shown in Table 2, thereby effectively mimicking the impact of hypoxia on the macromolecular composition of *P. pastoris *[41]. We therefore suggested that the strong transcriptional induction of the pathway under oxygen scarcity might only reflect compensation for the intermediate substrate deficit provoked by reduced oxygen availability. In which way ergosterol depletion affects protein secretion has to be elucidated, but combining published data with the outcome of this work points to the plasma membrane as key player. Ergosterol and sphingolipids are highly abundant in the yeast plasma membrane and to a lesser extent in other cellular membranes, where they assemble to form so-called lipid rafts [42]. These microdomains are composed of tightly packed sphingolipid acyl chains, attributing them detergent resistance (i.e. to non-ionic surfactants like Triton X-100). An important feature of such lipid rafts is their contribution to protein sorting, since the targeting of membrane protein cargo to the cell surface requires early raft association already in the ER [43]. As a consequence, defective synthesis of sphingolipids and ergosterol has been shown to impair the trafficking and sorting of a raft-associated chimeric protein to the cell surface [43], and also resulted in missorting of the plasma membrane ATPase Pma1p to the vacuole for degradation [44,45]. Bagnat and co-workers have shown that sphingolipids and ergosterol were required for incorporation of the cell wall protein Gas1p (a GPI-anchored α-1, 3-glucanosyltransglycosylase) to detergent resistant membranes (DRM), but not for vacuolar or secreted proteins [46]. Consequently, ergosterol depletion may lead to reduced Gas1p *in vivo *incorporation to the cell wall and, therefore, increased cell wall porosity due to reduced ß-glucan crosslinking; this effect might facilitate the passage of heterologous proteins through the cell wall in a similar way as observed in *GAS1 *knockout strains [47]. We assume that the defective transport of proteins destined to be incorporated to the plasma membrane might unbalance membrane fluidity by impairing the formation of lipid rafts. The consequences may include a less stringent control of the exchange of macromolecules between the cell and its proximate environment, possibly including secreted proteins whose translocation through the cell is not affected by ergosterol and sphingolipid depletion. It is also likely that membrane proteins that are not incorporated into the DRM, which usually takes place at the ER or Golgi level, are not incorporated into transport vesicles either, thus alleviating such transport compartments from cargo and facilitating the additional uptake and translocation of soluble proteins. These results, although preliminary, support evidence for a complex interaction between cellular membranes and protein secretion, implicating the plasma membrane as hitherto only marginally regarded, but promising target for strain engineering.

In addition, non-ionic surfactants including Tween80, Tween 20 and Triton X-100 were identified as potential enhancers of Fab secretion in *P. pastoris *with the highest (2 fold) increase in Fab productivity of cells grown in the presence of Tween 80 and a less pronounced increase after addition of Tween 20 or Triton X-100. This is not the first study that reports evidence for a stimulatory effect of Tweens or similar non-ionic surfactants on protein secretion. In bacteria, Tween 80-stimulated glycosyltransferase production correlated with alterations in membrane fluidity [26,27]. The authors of these studies suggested that an increase in fluidity of the membrane lipids might facilitate the release of intracellular accumulated protein, thereby enhancing its rate of secretion. The beneficial effect of surfactants, including Tween, appeared to be valid also in other organisms, including cellulase secreting *Trichoderma reesei *[48], recombinant *Schizosaccharomyces pombe *[49] and recently also in recombinant *P. pastoris *[50,51]. Apart from the more apparent explanation of a "leakier" plasma membrane that facilitates translocation of soluble proteins, speculations about the mechanisms underlying this effect also included fluctuations in the electrochemical gradient and enhanced membrane fusions of transport vesicles. This latter finding could also explain the observed increase in secreted Fab after the decrease of ergosterol biosynthesis by fluconazole treatment. A destabilization of the membranes by sterol deficiency could favour such membrane fusions and increase the volume of transport vesicles and consequently also the size of the cargo to be delivered to the surface.

In good accordance with these assumptions, in *S. cerevisiae *cultures grown under comparable conditions and expressing the same model protein, no hypoxic effect - i.e. favoured protein secretion by oxygen depletion - has been observed, probably because ergosterol synthesis did not seem to be affected on the transcriptional level either [52].

Our findings give therefore strong evidence for cellular membranes or membrane related genes and pathways as promising strain engineering targets.

## Conclusions

The current study illustrates how the combination of comprehensive genome-scale transcriptomics analyses using host specific DNA microarray technologies and physiological studies under defined conditions of different oxygen availability allowed for the identification of novel potential target genes and pathways for the engineering of the industrially important yeast *P. pastoris*. There is evidence that the complexity of many interacting elements (or cellular processes) makes metabolic engineering for improved protein secretion a challenging task. Nevertheless, previous reports clearly point out that the engineering of the protein folding and quality control system in the ER is a feasible strategy, even if it is only based on the modification of a single gene. Protein folding and ER quality control are certainly two of the best explored mechanisms in engineering studies and comprise a great number of target genes, essentially because of their apparent interrelation with protein production.

Even more promising results pointed to a strong interrelation of the ergosterol pathway and, thus, the plasma membrane, with the yeast secretion system, making it a novel target for systematic strain engineering studies. In hypoxic conditions, the ergosterol biosynthetic genes were strongly induced, but the cellular ergosterol content was decreased. Mimicking these conditions by specifically blocking ergosterol synthesis with the antifungal agent fluconazole indeed resulted in an increased Fab secretion. Furthermore, plasma membranes also undergo perturbations upon treatment with non-ionic surfactants like Tweens. The fact that treatment of *P. pastoris *with such detergents yielded in a higher amount of extracellular Fab provides further evidence for a close interrelation between protein secretion and plasma membrane alterations.

Altogether, it turned out that such a systematic approach for finding novel targets, albeit not being a high-throughput strategy, serves as valuable tool for strain engineering. Such a strategy would be even more powerful when applying similar cultivation conditions to other microbial host systems for a comparative analysis, with the aim to find host specific as well as common limitations in protein secretion. Moreover, we envisage that the integration of transcriptomics data into genome-scale metabolic models (made recently available for *P. pastoris*, [53]) should facilitate the systematic extraction of information useful for subsequent rational strain engineering strategies.

## Methods

### Strains and strain engineering

The starting strain used in this study was a *Pichia pastoris *X-33 pGAPZ*α*A Fab2F5 strain. A detailed description of the construction of the vector containing the recombinant protein as well as transformation conditions are found in [54]. In brief, the expression cassettes for the light and heavy chain of the human Fab 2F5 antibody fragment were separately placed under the control of the *P. pastoris *GAP (glyceraldehyde-3-phosphate dehydrogenase) promoter and combined on one plasmid. This vector, conferring resistance to Zeocin™, was integrated into the genome of *P. pastoris *host strain X-33 (wild-type phenotype).

Eight host specific gene targets have been selected for co-overexpression studies in the Fab 2F5 expressing X-33 *P. pastoris *strain. The selection of these genes was based on a previous genome-wide study in *P. pastoris *[22]. The list of genes and the corresponding primers used for the amplification from genomic X-33 DNA are given in Table 3. The primers were flanked with the sequences of the restriction sites SfiI and SbfI for cloning into the vector pPUZZLE [55], which confers resistance to kanamycin in bacteria and geneticin G418 in yeast. After propagation of the vectors carrying the gene of interest in *E. coli *DH5α, plasmids were purified and sequenced with the primers pGAP (5'GATTATTGGAAACCACCAGAATCG) and pPUZZLE (5'GGCGTGAATGTAAGCGTGAC). The verified plasmid constructs were then linearized with AscI (FastDigest, Fermentas) for integration into the transcription termination locus of AOX1 (AOX1 TT), and transformed into X33 pGAPZ*α*A Fab 2F5 competent cells. For the reference strain, the empty pPUZZLE vector was used.

**Table 3:** List of primers used for amplification of genes for co-expression in *P.pastoris *Fab 2F5

Target gene	Primer name	Primer sequence
**ERO1**	ERO1_fwd	**5' **AACTGCCTGCAGGACC_ATGAGGATAGTAAGGAGCGTAGCTAT
	ERO1_rev	**5'**AATCGGGCCGAGGCGGCC_TTACAAGTCTACTCTATATGTGGTATCT

**NCE103**	NCE103_fwd	**5' **AAGCGCCTGCAGGACC_ATGGGTGGTTTATCATTTGA
	NCE103_rev	**5' **AATCGGGCCGAGGCGGCC_TTAATGTCCACCGGCTTCAGTATCA

**AQR1**	QDR2_fwd	**5' **AACTGCCTGCAGGACC_ATGACAAATGAAAAATTGGATTTG
	QDR2_rev	**5'**AATCGGGCCGAGGCGGCC_CTACAGTTTGTATTTTGTTCCCCTCCTA

**SLY41**	SLY41_fwd	**5' **AACTGCCTGCAGGACC_ATGATCATTACGCAGAATCT
	SLY41_rev	**5' **AATCGGGCCGAGGCGGCC_CTAGTTTTTGACTGCACCCCATTT

**TDH3**	TDH3_fwd	**5' **AAGCGCCTGCAGGACC_ATGGCTATCACTGTCGGTAT
	TDH3_rev	**5' **AATCGGGCCGAGGCGGCC_TTAAGCCTTAGCAACGTGTT

**TEF4**	TEF4_fwd	**5' **AACTGCCTGCAGGACC_ATGTCGCAAGGAACAATTTAC
	TEF4_rev	**5' **AATCGGGCCGAGGCGGCC_TTAATTACTCTTGGGTGGAACAT

**TSA1**	TSA1_fwd	**5' **AACTGCCTGCAGGACC_ATGTTTGGACTAAATCACGAGATA
	TSA1_rev	**5' **AATCGGGCCGAGGCGGCC_CTATTTGGACTTGGAAAAGAA

**WSC4**	WSC4_fwd	**5' **AACTGCCTGCAGGACC_ATGTTGTTGAAGTTGATTTGGGTATTT
	WSC4_rev	**5' **AATCGGGCCGAGGCGGCC_TTAGGCATTATTTCCTGGGGTCTCT

List of primer sequences used for amplification of the target genes from genomic *P. pastoris *DNA http://www.pichiagenome.org. The sequences of the restriction sites SfiI (GGCC NNNN^NGGCC) and SbfI (CCTGCA^GG) are underlined.

### Transformation and direct selection of yeast transformants on G418

*Pichia pastoris *competent cells were prepared according to the condensed protocol described in [56]. Prior to transformation, 40 μl competent cells were mixed with 100 ng of the linearized and purified plasmid DNA and chilled on ice for 5 minutes. Electroporation was carried out on a BioRad Gene Pulser (BioRad), with the electroporation parameters set to 1500 V, 25 μF and 400 Ω. Shortly after transformation, cells were resuspended in 500 μl 1 M sorbitol and 500 μl YPD and incubated at 30°C for at least 3 h or overnight in 15 ml Falcon tubes. About 25 - 300 μl of this cell suspension was spread on YPD agar plates containing 500 μg ml^-1 ^G418 (Invivogen). After 2 - 3 days only the big (Ø > 2 mm) colonies were picked, and its genomic DNA extracted (Wizard^® ^Genomic DNA Purification Kit, Promega) and checked for correct integration by PCR screening with the pGAP and pPUZZLE primers (vector specific).

### Cultivation conditions and screening for Fab expression

For the screening in 24-well cell culture plates (Whatman), 2 ml of Buffered Minimal Glycerol (BMG) medium (100 mM potassium phosphate (pH 6.0), 1.34% (w/v) yeast nitrogen base (without amino acids), 4 × 10^-5 ^% (w/v) biotin, 1% (v/v) glycerol were inoculated with a fresh colony and incubated over night at 25°C and 250 rpm in an orbital shaker (Infors). Cultures were sealed with gas permeable, sterile BREATHseal™ (Greiner) membranes for maintaining optimal oxygen supply in the cultures. After 14-16 hours, optical density was measured at 600 nm (OD_600_), and cells were diluted into a fresh Buffered Minimal Dextrose medium (containing 2% (v/v) dextrose as carbon source) to an initial OD_600 _of 0.1. After 24 hours, 1 ml cells were harvested and centrifuged at 13, 000 rpm for 1 minute. The supernatant was stored at -20°C for ELISA analysis, and the pellet was used for the determination of the wet cell weight (WCW). For each strain construct, relative Fab yield was calculated from technical triplicates on biological duplicates.

For the shake flask cultures, 5 ml of BMG medium in 50 ml-Falcon tubes were inoculated to an initial OD_600 _of 0.1 from an overnight culture in YPD and incubated at 25°C and 180 rpm in a Multitron II orbital shaker (Infors). After approximately 16 hours, these seed cultures were used to inoculate 250 ml-volume baffled Erlenmeyer containing 25 ml of a fresh BMD medium at an initial OD_600 _of 0.1). Cultures were incubated for 24 hours under the same temperature and agitation conditions. For the determination of the dry cell weight (DCW), 2 × 10 ml of the cultures were washed twice with sterile ddH_2_O and filtered through pre-dried and pre-weighed glass fibre filters (0.7 μm pore size, Whatman). Filters containing the yeast biomass were then dried at 105°C for 24 h, cooled down in a desiccator and weighed. The Fab yield from shake flask experiments (including Tween and fluconazole treatments), was determined from three biological replicates analysed in technical triplicates.

### Treatment with Tween 80, and other non-ionic surfactants

For the *P. pastoris *cultures treated with Tween 80, Tween 20 and Triton X-100 these components were dissolved (v/v) in water and added to the culture medium BMD at final concentrations of 42 mg l^-1^. Additionally, cells were cultured separately without any treatment as negative controls.

### Fluconazole treatment

Fluconazole is an antifungal agent and interferes with fungal ergosterol synthesis. It specifically inhibits lanosterol C-14a demethylase, encoded by the yeast *ERG11 *gene. A fluconazole stock solution (100 μg ml^-1 ^in H_2_O) was added to the culture medium BMD at final concentrations ranging between 0 and 1.0 μg ml^-1^.

### Analysis of total sterols

Total sterol content was quantified as previously described [57]. Briefly, lyophilized cells (0.1 g) were treated for 20 min at 100°C in 0.5 N HCl and allowed to cool to room temperature. After addition of 3 g of KOH and 12.5 ml of methanol with pyrogallol (0.25 g l^-1^) and stigmasterol as internal standard (8 mg l^-1^), the mixture was saponified by incubation for 1.75 h at 70°C in a water bath. Sterols were extracted two times with n-hexan (20 ml), dried by rotation evaporator, resuspended in 2 ml chloroform and derivatized with MSTFA (N-Methyl-N-trimethylsilyltrifluor-acetate). Sterols were separated on an Agilent 6890N gas chromatograph coupled with an Agilent 5975B VL mass spectrometer (GC-MS) on a capillary column (30 m by 0.25 mm and 0.25 μm film thickness; Agilent Technologies, HP-5MS, 19091S-433). The temperature was initially 150°C for 0.5 min; it was then increased at 40°C min^-1 ^to a temperature of 280°C, further increased at 2°C min^-1 ^to a temperature of 310°C and finally to a temperature of 350°C which was held for 2 min. The linear velocity was 19 cm s^-1^, helium was used as the carrier gas, and injections were run in the splitless mode. The injection volume was 1 μl. It was detected each mass between 29 to 500 amu all the time. The area of each peak was calculated and related to 1 g of cell dry weight. Each sample was measured in triplicate. Sterols were quantified via the internal standard stigmasterol and the external standards ergosterol, cholesterol and squalene. Ergosterol was used for the quantification of all "ergosta"-Intermediates as well as for substances which could not be identified. Cholesterol was used for the quantification of all "cholesta"-intermediates and squalene was used for the quantification of squalene.

### Quantification of the Fab 2F5 antibody by sandwich ELISA

96 well plates (Nunc) were pre-coated with a Fab-specific anti-human IgG (Sigma) 1:1000 diluted in PBS, and incubated over night at room temperature. After washing the plates, samples and the Fab standard (Bethyl Inc.) were diluted in PBS containing 10% (w/v) bovine serum albumin and 0.1% (v/v) Tween 80 and applied in duplicates (standard) or triplicates (samples). Plates were incubated for 2 hours, washed, and incubated for another hour with the secondary alkaline phosphatase-conjugated anti-kappa light chain antibody (Sigma). After a thorough washing step, plates were treated with the phosphatase substrate pNPP (Sigma) and measured at 405 nm (reference filter 620 nm) on a micro plate reader (Fisher Scientific). Data analysis was performed by using a standard curve (hFab standard) and a polynomial function.

### Genomic DNA and total RNA preparation

Selected clones overexpressing the genes of *Ero1 *and *Wsc4*, as well as the control strain (X-33 producing the Fab 2F5) were cultured in BMD (2% Dextrose) media to an initial OD_600 _of 0.1 for 24 h at 25°C and 180 rpm. To maintain cells for further extractions, 9 ml of culture were mixed with 5 ml of freshly made chilled 5% (v/v) phenol (Sigma) solution in absolute ethanol, centrifuged at 4°C and 12, 000 rpm for 5 min; harvested cells were stored at -80°C until extraction.

RNA extractions were performed with RNeasy Mini Kit (Quiagen) following manufacturer's protocol of enzymatic extraction using lyticase (Sigma). RNA samples were quantified and analysed for purity using Experion RNA StdSens Analysis Kit (Bio-Rad) with a RQI between 8.8 and 9.9.

Genomic DNA extraction of the cells was also performed using Wizard^® ^SV Genomic DNA (Promega) and quantified with Nanodrop™3300 (Thermo Scientific). Samples were diluted in DEPC water (Ambion) to a final concentration of 1 ng μl^-1 ^to be further used in qRT-PCR.

### cDNA generation and primer design

For the generation of cDNA, RNA extractions were subjected to a DNAse I Amplification Grade (Invitrogen) treatment prior to reverse transcription with SuperScript^®^VILO cDNA Synthesis Kit (Invitrogen). All steps were performed following the manufacturer's protocol, starting from 1 μg RNA. After cDNA generation, samples were purified (Clean-Up Wizard^® ^SV Gel and PCR Clean up system, Promega) and quantified with Nanodrop™3300 (Thermo Scientific). Samples were diluted with DEPC water (Ambion) to a final concentration of 1 ng μl^-1 ^and 2 μl were used for qRT-PCR analysis. Oligonucleotides (purchased from biomers.net) were designed with the Clone Manager Professional, version 9 software http://www.scied.com and Primer3 http://frodo.wi.mit.edu/primer3/, considering an amplicon size of 100 - 200 bp and a Tm of approximately 60°C (Table 4).

**Table 4:** List of primers used for quantification of gene dosage and transcriptional levels by qRT-PCR

Target gene	Primer name	Primer sequence
**ERO1**	5'ERO1_qPCR_vie	**5' **GTTGGAAAAGCCGCATATAAACAAAACA
	3'ERO1_qPCR_vie	**5' **CAGCTTGGGCAAAGTCCTGTAAGAGTTC

**WSC4**	5'WSC4_qPCR	**5' **CAGCACCATCCATATCAACC
	3'WSC4_qPCR	**5' **GTTTGCGGATCTTGAGCTAC

**ACT1**	5'ACT1_qPCR_vie	**5' **CCTGAGGCTTTGTTCCACCCATCT
	3'ACT1_qPCR_vie	**5' **GGAACATAGTAGTACCACCGGACATAACGA

**2F5_HC**	5'2F5_HC_qPCR	**5' **CTCTCACGCTGACCTGTTCC
	3'2F5_HC_qPCR	**5' **GATTGCAAGCCACTCTAGGG

**2F5_LC**	5'2F5_LC_qPCR	**5' **CTTCCCGCCATCTGATGAGC
	3'2F5_LC_qPCR	**5' **GAGGGCGTTATCCACCTTCC

List of primer sequences used for qRT-PCR assays with both genomic DNA and cDNA.

### Primer validation and amplicon purification for standard curve

To guarantee that each primer pair yields a single PCR product of the predicted size, we performed a conventional PCR and confirmed the absence of any primer dimers or unspecific products on a 2% (w/v) agarose gel. To additionally check the specificity of the assay, a melt-curve analysis was performed at the end of each PCR assay. An optimized reaction should have a single peak in the melt-curve, corresponding to the single band on the agarose gel. The specific PCR products were purified (Wizard^® ^SV Gel and PCR Clean-Up System, Promega) and quantified on a Nanodrop™3300 (Thermo Scientific). From the concentration and the size of the amplicon, the copy number per μl was determined according to Whelan [58] and decimal dilutions representing 10^7 ^- 10^3 ^copies of target DNA were used for standard curve generation.

### qRT-PCR assay

Quantitative real-time PCR was carried out in 20 μl reactions using semi-skirted iQ 96-well PCR plates and iQ™SYBR^® ^Green supermix (Bio-Rad). Samples were measured in triplicates and standards were measured in duplicates on the iCycler Thermal Cycler (Bio-Rad). A non-template control was run in every experiment for each of the primer pairs to avoid detection of unspecific priming. The reactions were incubated at 95°C for 10 min to activate the *Taq *polymerase, and then subjected to a three-step cycling protocol including melting (95°C, 15 sec), annealing (58°C, 15 sec) and extension (72°C, 30 sec) for a total of 40 cycles. Each extension was followed by data collection at 72°C. After a final extension of 5 min at 72°C, a melt-curve profile was generated by data collection during 81 cycles starting at 55°C to 95°C, with 0.5°C increments/cycle (1-sec intervals).

### Data analysis

The relative gene expression was calculated for each sample with three measurements giving a maximum standard deviation of 1%. Since amplification efficiencies of the target and reference genes were not the same, Pfaffl method [59] was chosen for the relative quantification of qRT-PCR results.

## Competing interests

The authors declare that they have no competing interests.

## Authors' contributions

KB carried out microarray data mining for the identification of the target genes and potential target pathways, designed the experiments, carried out the cloning and screening of yeast transformants, participated in the cultivation experiments and quantification of product titers, and drafted the manuscript. NA carried out the cultivation experiments and quantification of the product titers and biomass. DM participated in designing the study, data interpretation and manuscript revision. CL participated in the sterol analyses and data interpretation, and manuscript revision. PF conceived this study, participated in the interpretation of results and assisted manuscript drafting. All authors read and approved the final version of the manuscript.

## Supplementary Material

Additional file 1 **Sterol profiling of *P. pastoris *cells by GC analysis**. Sterol composition profile of *P. pastoris *X-33 pGAPZαA Fab2F5 cells treated with 0.6 μg ml^-1 ^fluconazole and without treatment (control) was determined by GC-MS. Chromatograms showing ergosterol depletion and the appearance of two new unknown sterol peaks with a longer retention time than ergosterol in fluconazole-treated cultures are represented. Click here for file
