# Supplementary figures and images for: Protein trafficking, ergosterol biosynthesis and membrane physics impact recombinant protein secretion in Pichia pastoris
Source: Microb Cell Fact. 2011 Nov 3;10:93. doi: 10.1186/1475-2859-10-93 (PMC3219557; doi:10.1186/1475-2859-10-93)

## Slide 1
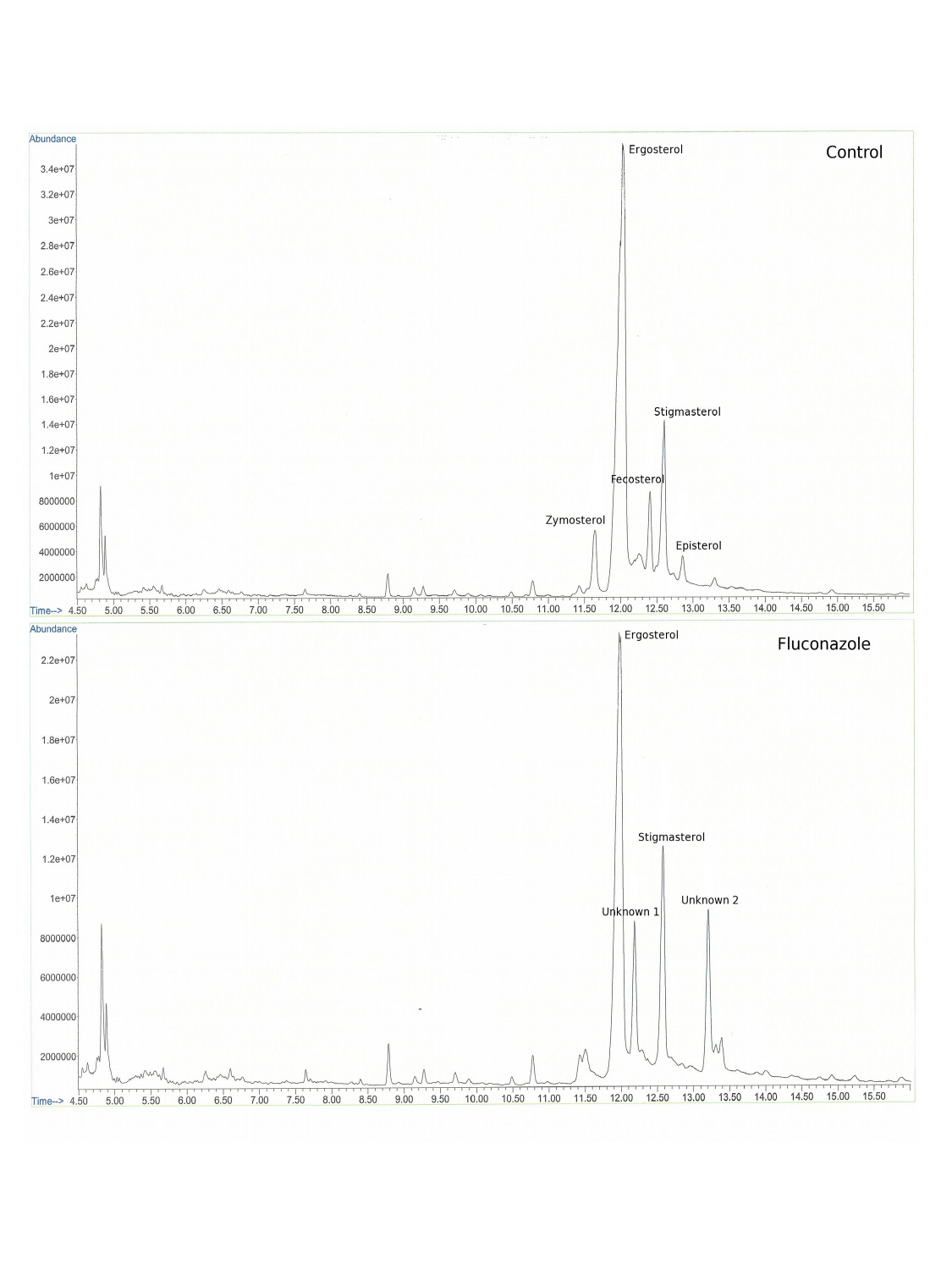

Supplement: Additional file 1 — Sterol profiling of P. pastoris cells by GC analysis. Sterol composition profile of P. pastoris X-33 pGAPZαA Fab2F5 cells treated with 0.6 μg ml-1 fluconazole and without treatment (control) was determined by GC-MS. Chromatograms showing ergosterol depletion and the appearance of two new unknown sterol peaks with a longer retention time than ergosterol in fluconazole-treated cultures are represented. [file 1475-2859-10-93-S1.PPT]
